#  Sociodemographic inequalities in sleep duration and quality at 11, 18, and 22 years of age: a cross-sectional study using data from the 1993 Pelotas Birth Cohort, 2004-2015 

**DOI:** 10.1590/S2237-96222026v35e20260212.en

**Published:** 2026-08-05

**Authors:** Fernando Cesar Wehrmeister, Adriana Kramer Fiala Machado, Angel Caroline Chirivino Antunes da Rocha, Indiara da Silva Viegas, Larissa Adna Neves Silva, Michele Krüger Vaz Moreira, Tainã Dutra Valério, Helen Gonçalves, Ana Maria Baptista Menezes

**Affiliations:** 1 Programa de Pós-graduação em Epidemiologia da Universidade Federal de Pelotas, RS, Brasil Universidade Federal de Pelotas Programa de Pós-graduação em Epidemiologia Universidade Federal de Pelotas RS Brazil; 2 Programa de Pós-graduação em Epidemiologia da Universidade Federal de Pelotas, RS, Brasil Universidade Federal de Pelotas Programa de Pós-graduação em Epidemiologia Universidade Federal de Pelotas RS Brazil; 3 Programa de Pós-graduação em Epidemiologia da Universidade Federal de Pelotas, RS, Brasil Universidade Federal de Pelotas Programa de Pós-graduação em Epidemiologia Universidade Federal de Pelotas RS Brazil; 4 Programa de Pós-graduação em Epidemiologia da Universidade Federal de Pelotas, RS, Brasil Universidade Federal de Pelotas Programa de Pós-graduação em Epidemiologia Universidade Federal de Pelotas RS Brazil; 5 Programa de Pós-graduação em Epidemiologia da Universidade Federal de Pelotas, RS, Brasil Universidade Federal de Pelotas Programa de Pós-graduação em Epidemiologia Universidade Federal de Pelotas RS Brazil; 6 Programa de Pós-graduação em Epidemiologia da Universidade Federal de Pelotas, RS, Brasil Universidade Federal de Pelotas Programa de Pós-graduação em Epidemiologia Universidade Federal de Pelotas RS Brazil; 7 Programa de Pós-graduação em Epidemiologia da Universidade Federal de Pelotas, RS, Brasil Universidade Federal de Pelotas Programa de Pós-graduação em Epidemiologia Universidade Federal de Pelotas RS Brazil; 8 Programa de Pós-graduação em Epidemiologia da Universidade Federal de Pelotas, RS, Brasil Universidade Federal de Pelotas Programa de Pós-graduação em Epidemiologia Universidade Federal de Pelotas RS Brazil; 9 Programa de Pós-graduação em Epidemiologia da Universidade Federal de Pelotas, RS, Brasil Universidade Federal de Pelotas Programa de Pós-graduação em Epidemiologia Universidade Federal de Pelotas RS Brazil

**Keywords:** Health inequalities, Sleep, Sleep Quality, Socioeconomic Factors, Cross-Sectional Studies., Disparidades en el Estado de Salud, Sueño, Calidad del Sueño, Factores Socioeconómicos, Estudios Transversales.

## Abstract

Objective: To analyze sleep duration and quality at different ages according to sociodemographic factors.

Methods: This was a cross-sectional study using data from the 1993 Pelotas Birth Cohort in Rio Grande do Sul, Brazil, with follow-ups conducted at ages 11, 18, and 22. Sleep duration and quality were self-reported; daily sleep duration was estimated from weekday bedtime and wake-up times, and sleep quality was assessed at 22 years of age using a question about sleep perception. Descriptive analyses were performed, and inequality measures were estimated according to sex, race/skin color, and wealth index.

Results: A total of 4,439, 4,081, and 3,800 individuals were analyzed at ages 11, 18, and 22, respectively. Mean sleep duration was 9.7 (95% confidence interval [95%CI] 9.6; 9.7), 8.4 (95%CI 8.3; 8.5), and 8.0 (95%CI 7.9; 8.0) hours. The absolute inequality index at 18 years of age was -1.3 (95%CI -1.5; -1.0) among females and -0.7 (95%CI -1.0; -0.4) among males. At 22 years of age, the proportion reporting “very good” sleep quality was 19.2% (95%CI 17.4; 21.1) among males, while “very poor” sleep quality was 5.2% (95%CI 4.3; 6.2) among females.

Conclusion: Sleep duration decreased between 11 and 22 years of age and was longer among females and more vulnerable groups, among whom very good sleep quality was less frequent.

## Resumen

Objetivo: Analizar la duración y la calidad del sueño en diferentes edades según factores sociodemográficos.

Métodos: Estudio transversal con datos de la Cohorte de Nacimientos de 1993 de Pelotas, Rio Grande do Sul, con mediciones a los 11, 18 y 22 años. La duración y la calidad del sueño fueron autorreportadas; la duración diaria se estimó a partir de los horarios de acostarse y levantarse en días laborables, y la calidad se evaluó a los 22 años mediante una pregunta sobre la percepción del sueño. Se realizaron análisis descriptivos y se estimaron medidas de desigualdad según sexo, raza/color e índice de bienes.

Resultados: Se analizaron 4.439, 4.081 y 3.800 individuos a los 11, 18 y 22 años, respectivamente. La media de la duración del sueño fue de 9,7 (intervalo de confianza del 95% - IC95% 9,6; 9,7), 8,4 (IC95% 8,3; 8,5) y 8,0 (IC95% 7,9; 8,0) horas. El índice absoluto de desigualdad a los 18 años fue de -1,3 (IC95% -1,5; -1,0) en mujeres y de -0,7 (IC95% -1,0; -0,4) en hombres. A los 22 años, la proporción de calidad del sueño “muy buena” fue del 19,2% (IC95% 17,4; 21,1) en hombres y la de “muy mala” fue del 5,2% (IC95% 4,3; 6,2) en mujeres.

Conclusión: La duración del sueño disminuyó entre los 11 y los 22 años y fue mayor entre mujeres y en grupos más vulnerables, en los que la calidad del sueño “muy buena” fue menos frecuente.

**Table:** Ethical aspects

This research respected ethical principles, having obtained the following approval data:		
Research ethics committee	Número do parecer	Approval date
Universidade Federal de Pelotas	Protocol No. 29/2003 (11 years of age)	1/12/2003
Universidade Federal de Pelotas	Protocol No. 5/2011 (18 years of age)	12/10/2010		
Universidade Federal de Pelotas	1,250,366 (22 years of age)	29/9/2015
**Informed consent record**	Obtained from all participants. Informed consent was obtained from all participants or from their parents, when under 18 years of age.	

## Introduction

Changes in sleep duration and quality are recognized as relevant public health problems and directly affect individuals’ physical and mental well-being [1]. Sleep duration plays an important role in maintaining health and influences mechanisms related to energy balance and hormonal production, with repercussions in both adolescence and adulthood [2]. Inadequate sleep is associated with negative health outcomes, including obesity, hypertension, and mental health problems such as depression and bipolar disorder [3,4].

Throughout the life course, there is a tendency toward reduced required sleep duration. Eight to ten hours of sleep are recommended for adolescents, and seven to nine hours for adults [5]. However, evidence from population-based studies between 2018 and 2023 indicated that a substantial proportion did not meet these recommendations, ranging from 66.0% among adolescents to 55.5% among adults aged 20 years or older, reflecting chronic sleep restriction that broadly affects different age groups [6,7]. Among adults, there is also a trend toward reduced sleep duration over the years, particularly among males [8].

During adolescence, sleep duration may have a protective effect against poor sleep quality. In contrast, delayed sleep onset and prolonged sleep latency may be associated with a higher likelihood of reporting nonrestorative sleep and dissatisfaction with sleep [6]. In adulthood, sleep durations closer to recommended levels contribute to better cognitive function and a reduced risk of chronic and neurodegenerative diseases [2,9].

In addition to age-related variations, sociodemographic factors have also been associated with sleep duration [10,11]. Overall, women sleep longer and report poorer sleep quality than men [10]. Regarding socioeconomic status, Brazilian adults with higher purchasing power tend to sleep less and report better sleep quality than those with lower socioeconomic status [12]. In contrast, in high-income countries, wealthier individuals have longer sleep duration and better sleep quality [13]. In the Brazilian context, data on these associations using standardized measures remain scarce, especially across different age groups and over time [14]. This highlights the importance of advancing knowledge of the association between sociodemographic characteristics and sleep duration and quality across different stages of the life course.

Using data from the 1993 Pelotas Birth Cohort in Rio Grande do Sul, Brazil, this study aimed to analyze sleep duration and quality across different ages by sociodemographic factors.

## Methods

### Study design

This is a cross-sectional study using data from the 1993 Pelotas Birth Cohort in Pelotas, Rio Grande do Sul, a medium-sized municipality in southern Brazil. Analyses were performed separately at ages 11, 18, and 22.

### Setting and participants

The 1993 Pelotas Birth Cohort included 5,249 individuals in the perinatal phase, representing 99.7% of all live births (5,265) to mothers residing in the urban area of Pelotas between 1 January and 31 December, 1993. This study used information collected at the 11-, 18-, and 22-year follow-ups, conducted in 2004, 2011, and 2015, with follow-up rates of 87.5%, 81.4%, and 76.3%, respectively. More detailed information on the cohort follow-up methodology can be found in other publications [15,16].

The sample size corresponded to the number of participants available at each follow-up (11, 18, and 22 years of age), considering only individuals with complete information on sleep duration and quality variables at each stage. The total number of participants interviewed at ages 11, 18, and 22 was 4,452, 4,106, and 3,810, respectively. After excluding participants with missing information (13, 25, and 10), the analyses included 4,439, 4,081, and 3,800 individuals, respectively. At 18 and 22 years of age, 31 (0.8%) and 43 (1.1%) participants were pregnant.

### Data sources and measurement

#### Outcome

Sleep duration was defined as the average daily number of self-reported hours of sleep on weekdays (excluding weekends) at ages 11, 18, and 22. In all three follow-ups, sleep duration was assessed using the following questions: “Usually, at what time do you go to sleep on a weekday, excluding Saturday and Sunday?” and “Usually, at what time do you wake up on a weekday, excluding Saturday and Sunday?”. Sleep duration was calculated as the difference between self-reported wake-up and bedtime during weekdays and was treated as a continuous variable (hours).

Subjective sleep quality was assessed only at 22 years of age, based on overall sleep perception in the previous month, categorized as very good, good, poor, or very poor.

#### Independent variables

The independent variables assessed were: (i) sex (male, female), collected at baseline; (ii) race/skin color, self-reported at 15 years of age (White, Black, and Brown [Brazilian mixed race]); and (iii) wealth index (quintiles), collected at 11, 18, and 22 years of age.

The wealth index was based on principal component analysis of selected assets and household characteristics and was subsequently divided into quintiles [17]. The first quintile (Q1) corresponds to the poorest 20% of the population, whereas the last quintile (Q5) corresponds to the richest 20%. Race/skin color was categorized according to the criteria of the Brazilian Institute of Geography and Statistics (Instituto Brasileiro de Geografia e Estatística, IBGE) (White, Brown, Black, Asian, and Indigenous). Due to the small sample size (3.7%), individuals self-identified as Asian or Indigenous were excluded from the analysis.

### Statistical analysis

Analyses were conducted using Stata software, version 18.0 (Stata Corporation, College Station, United States). Sample description was performed using the proportions of individuals in each category of sociodemographic variables, accompanied by 95% confidence intervals (95%CI). Comparisons of proportions between the analytical sample and the cohort baseline (perinatal) sample were conducted by sex, race/skin color, and family income to assess potential selection bias. Family income was used in this comparison because baseline wealth index data were unavailable.

Mean sleep duration was reported separately by sex, wealth index, and race/skin color at ages 11, 18, and 22 years. Student’s t-test and analysis of variance (ANOVA) were used, and for the wealth index variable, a test for linear trend was applied.

To assess potential interactions in sleep duration, linear regression models including interaction terms between sex and race/skin color, and between sex and wealth index, were used at each age. The significance of interaction terms was assessed using Wald tests (Supplementary Table 1).

Given evidence of interaction, analyses were conducted with double stratification by: (i) sex and wealth index; and (ii) sex and race/skin color. To assess inequalities, the slope index of inequality (SII) was calculated for the wealth index variable [18]. For the double stratification using race/skin color, the mean absolute difference from the mean (MADM) was calculated. For this inequality measure, the standard error was estimated using a resampling technique, allowing the construction of confidence intervals [19].

## Results

The sample was predominantly composed of females across the three follow-ups (50.9%, 50.9%, and 53.2% at 11, 18, and 22 years, respectively) and of participants who self-identified as White. At 11 and 22 years of age, the distribution of family income differed from that in the perinatal period (p-values 0.030 and

<0.001). This difference was mainly explained by a lower proportion of individuals in the second quintile (23.3% in the perinatal period and 20.7% and 20.2% at 11 and 22 years, respectively), while the remaining quintiles showed similar values (Supplementary Table 2).

Overall mean sleep duration decreased with age. At 11 years, the mean was 9.7 hours (95%CI 9.6; 9.7), decreasing to 8.4 hours (95%CI 8.3; 8.5) at 18 years and to 8.0 hours (95%CI 7.9; 8.0) at 22 years.

Females had higher mean sleep duration than males in the two later follow-ups (8.8 versus 8.0 at 18 years [p-value <0.001] and 8.3 vs. 7.6 at 22 years [p-value <0.001]). At 18 and 22 years, individuals self-identified as White showed lower mean sleep duration (18 years: 8.3; 22 years: 7.9) compared to Black individuals (18 years: 8.7; 22 years: 8.1) and Brown individuals (18 years: 8.6; 22 years: 8.2) (Table 1). It was also observed that the higher the socioeconomic level, the shorter the sleep duration (p-value < 0.001). In the poorest quintile, mean sleep duration decreased with age: 9.8 (95%CI 9.7; 9.9) at 11 years, 9.0 (95%CI 8.9; 9.1) at 18 years, and 8.4 (95%CI 8.2; 8.5) at 22 years. In the richest quintile, mean sleep duration also decreased: 9.4 (95%CI 9.4; 9.5) hours at 11 years, 8.0 (95%CI 7.9; 8.1) hours at 18 years, and 7.8 (95%CI 7.6; 7.9) hours at 22 years (Table 1).

At all ages and for both sexes, an inverse relationship was observed between sleep duration and wealth quintiles, particularly among females from 18 years onward (Figure 1). At that same age, the absolute inequality index was -1.3 hours (95%CI -1.5; -1.0) among females and -0.7 hours (95%CI -1.0; -0.4) among males. Inequality measures (mean absolute difference from the mean) indicated that white females had lower mean sleep duration, with mean differences of 0.16 (95%CI 0.08; 0.23) at 18 years

and 0.18 (95%CI 0.11; 0.26) at 22 years relative to the mean (Figure 2).

The prevalence of sleep quality categories at 22 years was presented, stratified by sex, race/skin color, and wealth index (Table 2). “Very good” sleep quality was more frequent among males (19.2%; 95%CI 17.4; 21.1), while the “very poor” category was more prevalent among females (5.2%; 95%CI 4.3; 6.2). Individuals self-identified as White showed a higher prevalence of “very good” sleep quality (19.8%; 95%CI 18.2; 21.5) compared to those self-identified as Brown (14.0%; 95%CI 11.5; 17.0). Prevalences were similar across the remaining sleep quality categories.

Regarding the wealth index, the prevalence of “very good” sleep quality was 14.6% (95%CI 12.3; 17.3) in the lowest quintile and 20.3% (95%CI 17.6; 23.3) in the highest quintile, whereas the “very poor” category showed prevalences of 4.9% (95%CI 3.6; 6.7) and 3.0% (95%CI 2.0; 4.5), respectively. The remaining categories showed similar values across groups, with overlapping confidence intervals (Table 2).

**Table 1:** Sample description, mean sleep duration, and 95% confidence interval (95%CI) according to sex, race/skin color, and wealth index at 11, 18, and 22 years of age. Pelotas, 2004 (n=4,439), 2011 (n=4,081), and 2015 (n=3,800)

11 years old (n=4,439)				18 years old (n=4,081)			22 years old (n=3,800)		
Variables	n (%)	Mean (95%CI)	p-value	n (%)	Mean (95%CI)	p-value	n (%)	Mean (95%CI)	p-value
**Overall**		9,7 (9,6; 9,7)			8,4 (8,3; 8,4)			8,0 (7,9; 8,0)	
**Sex**	0,008^b^			<0,001^b^			<0,001^b^		
**Male**	2.180 (49,1)	9,6 (9,6; 9,7)		2.004 (49,1)	8,0 (8,0; 8,1)		1.778 (46,8)	7,6 (7,5; 7,7)	
**Female**	2.259 (50,9)	9,7 (9,7; 9,8)		2.077 (50,9)	8,8 (8,7; 8,8)		2022 (53,2)	8,3 (8,2; 8,4)	
**Race/skin color**^a^	0,944^c^			<0,001^c^			<0,001^b^		
White	2.704 (63,9)	9,7 (9,6; 9,7)		2.512 (64,2)	8,3 (8,2; 8,4)		2.256 (63,3)	7,9 (7,8; 8,0)	
Black	601 (14,2)	9,7 (9,6; 9,8)		565 (14,4)	8,7 (8,5; 8,8)		536 (15,0)	8,1 (8,0; 8,3)	
Brown (Brazilian mixed race)	768 (18,2)	9,7 (9,6; 9,8)		691 (17,7)	8,6 (8,5; 8,7)		635 (17,8)	8,2 (8,1; 8,3)	
**Wealth Index (quintiles)**	<0,001^d^			<0,001^d^			<0,001^d^		
Q1 (poorest)	859 (20,1)	9,8 (9,7; 9,9)		831 (20,4)	9,0 (8,9; 9,1)		759 (20,0)	8,4 (8,2; 8,5)	
Q2	851 (19,9)	9,8 (9,7; 9,9)		797 (19,5)	8,5 (8,4; 8,6)		759 (20,0)	8,1 (7,9; 8,5)	
Q3	853 (20,0)	9,7 (9,6; 9,8)		816 (20,0)	8,3 (8,2; 8,4)		759 (20,0)	8,0 (7,8; 8,1)	
Q4	854 (20,0)	9,6 (9,5; 9,7)		820 (20,1)	8,2 (8,1; 8,3)		758 (20,0)	7,8 (7,7; 7,9)	
Q5 (richest)	856 (20,0)	9,4 (9,4; 9,5)		814 (20,0)	8,0 (7,9; 8,1)		759 (20,0)	7,8 (7,6; 7,9)	

^a^Variable with the highest number of missing values. Individuals self-identified as yellow or Indigenous were excluded due to sample size; ^b^Student’s t-test; ^c^Analysis of variance (ANOVA) test; ^d^Test for linear trend.

**Figure 1:**
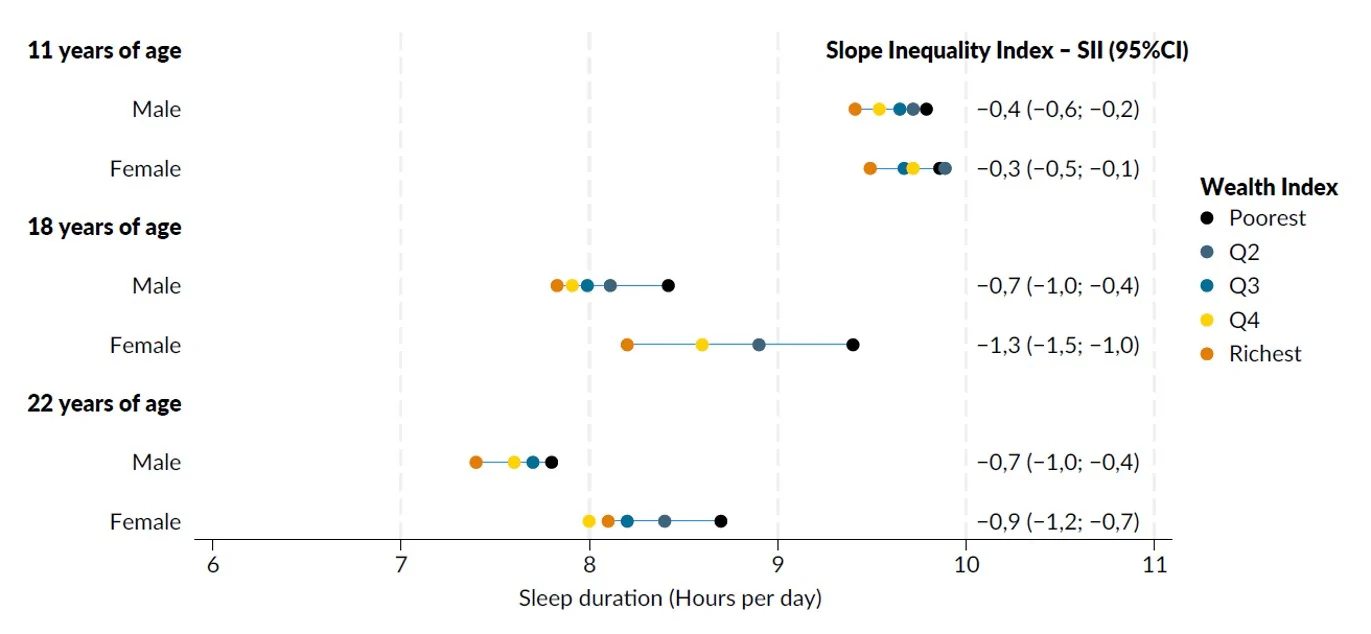
Mean sleep duration (hours), slope index of inequality (SII), and 95% confidence interval (95%CI) according to sex and wealth index at 11, 18, and 22 years of age. Pelotas, 2004 (n=4,439), 2011 (n=4,081), and 2015 (n=3,800)

**Figure 2:**
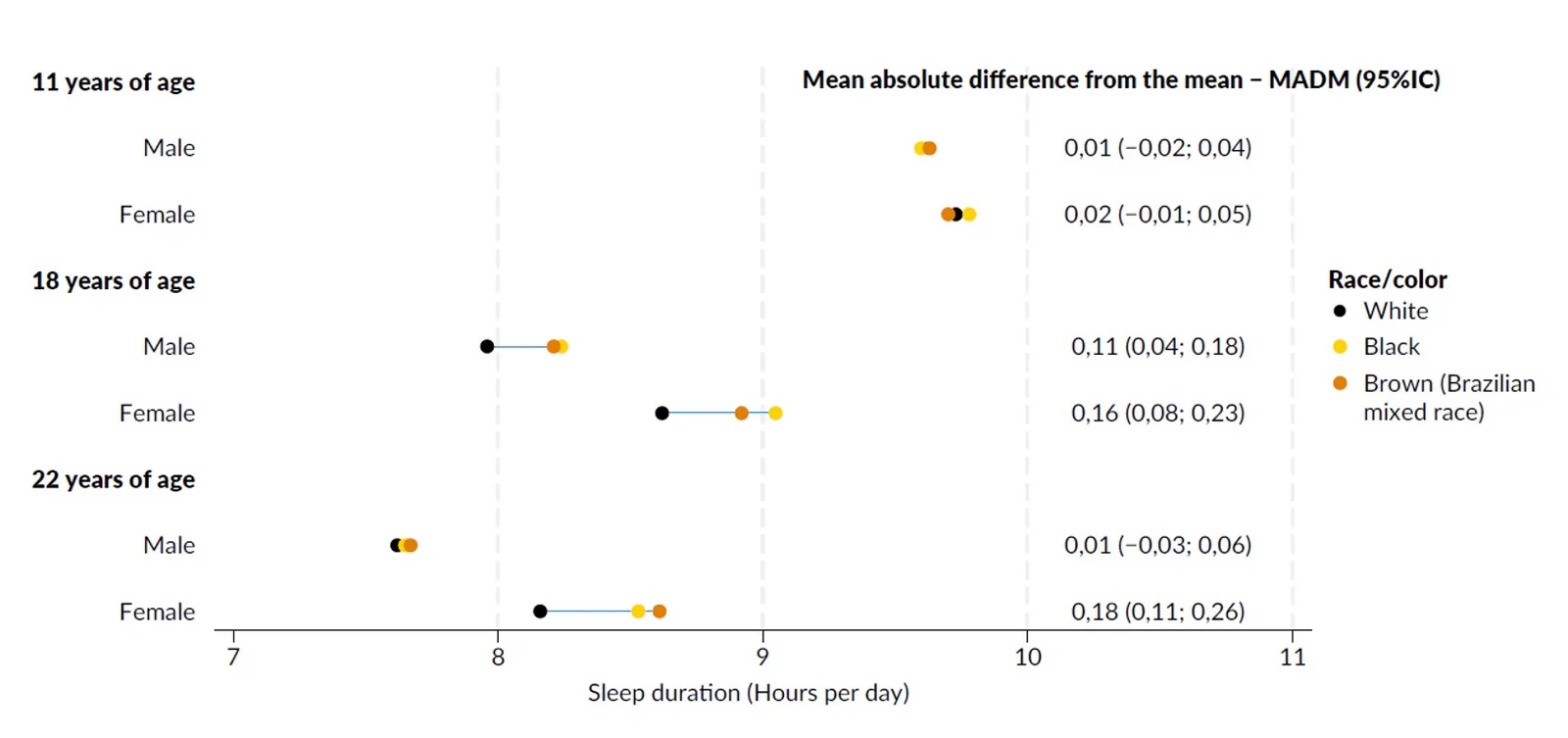
Mean sleep duration (hours), mean absolute difference from the mean (MADM), and 95% confidence interval (95%CI) by sex and race/skin color at 11, 18, and 22 years of age. Pelotas, 2004 (n=4,439), 2011 (n=4,081), and 2015 (n=3,800)

**Table 2:** Prevalence and 95% confidence interval (95%CI) of sleep quality categorized as “very good,” “good,” “poor,” and “very poor” according to sex, race/skin color, and wealth index at 22 years of age. Pelotas, 2015 (n=3,800)

Variables	Very good		Good		Poor		Very poor	
	%	95%CI	%	95%CI	%	95%CI	%	95%CI
**Sex**								
Male	19,2	17,4; 21.1	65,2	63,0; 67,4	12,6	11,2; 14,2	3,0	2,3; 3,9
Female	17,3	15,7; 19,0	61,7	59,5; 63,8	15,9	14,3; 17,5	5,2	4,3; 6,2
**Race/skin color**								
White	19,8	18,2; 21,5	62,0	59,9; 64,0	14,2	12,8; 15,7	4,0	3,3; 4,9
Black	15,3	12,5; 18,6	65,7	61,5; 69,6	14,2	11,5; 17,4	4,9	3,3; 7,0
Brown (Brazilian mixed race)	14,0	11,5; 17,0	66,7	63,0; 70,3	15,5	12,8; 18,5	3,8	2,5; 5,6
**Wealth Index (quintiles)**								
Q1 (poorest)	14,6	12,3; 17,3	66,5	63,1; 69,8	14,0	11,7; 16,6	4,9	3,6; 6,7
Q2	17,8	15,2; 20,7	62,5	58,9; 65,8	14,6	12,3; 17,3	5,1	3,8; 7,0
Q3	18,6	16,0; 21,5	62,8	59,3; 66,2	14,5	12,2; 17,2	4,1	2,9; 5,7
Q4	19,4	16,8; 22,4	62,4	58,8; 65,7	14,5	12,2; 17,2	3,7	2,6; 5,3
Q5 (richest)	20,3	17,6; 23,3	62,5	59,0; 65,9	14,1	11,8; 16,8	3,0	2,0; 4,5

## Discussion

Consistent patterns of sociodemographic inequalities in sleep duration and quality were observed, with shorter sleep duration at older ages and longer sleep duration among females, among Black or Brown individuals, and among those with lower socioeconomic status. These patterns were more evident at ages 18 and 22. In contrast, higher socioeconomic levels were associated with shorter sleep duration, particularly among 18-year-old females, whereas differences according to race/skin color were more pronounced among adult females.

The interpretation of these findings should consider certain limitations. The estimation of sleep duration based on self-report may have introduced information bias, leading to possible overestimation, especially because it did not account for sleep latency and relied solely on bedtime and wake-up times. In this sense, participants may have interpreted “going to sleep” as the time of going to bed and “waking up” as the time of getting out of bed, which may have inflated the estimated total sleep time.

The assessment restricted to weekdays may also fail to reflect variations in sleep patterns on weekends. The lack of other sleep dimensions, such as latency, variability, and sleepiness, and of contextual factors such as stress and housing conditions, family responsibilities, and entry into higher education, limits a more comprehensive understanding of the mechanisms involved. The unavailability of variables such as physical activity level and overweight may limit understanding of these mechanisms, as they may serve as intermediate stages in the relationship between sociodemographic factors and sleep.

The lack of consistent assessment of sleep quality across follow-up waves, unlike sleep duration, hinders comparisons across the different ages evaluated and may obscure relevant changes in this outcome. Although the combination of quantitative and qualitative measures broadens the assessment perspective, these limitations should be considered when interpreting the results, as they may underestimate the complexity of sleep inequalities. Finally, the exclusion of minority ethnic-racial groups due to a small sample size may limit a more comprehensive analysis of inequalities.

The use of international references based predominantly on evidence from high-income countries constitutes a limitation, as these may not adequately reflect the dynamics of low- and middle-income populations [10]. However, the continuous measurement of sleep duration adopted in this study allows a more sensitive assessment of differences between social groups.

The observed patterns can be understood in light of the literature describing changes in sleep across development and the combined influence of biological, behavioral, and social factors. The reduction in sleep duration between ages 11 and 18 reflects a well-documented global trend, influenced by both behavioral factors and biological changes during puberty [20].

Circadian phase delay, characterized by delayed melatonin secretion, shifts the optimal sleep window to later times, making it difficult for adolescents to fall asleep early [2,20]. In this age group, this misalignment between biology and social demands, such as school schedules, leads to earlier wake-ups and contributes to chronic sleep deprivation. The nighttime use of electronic devices intensifies this pattern by delaying circadian rhythms and exacerbating sleep irregularity [21].

Between ages 18 and 22, sleep duration becomes more heterogeneous. It may range from slight recovery (due to greater schedule flexibility) to marked sleep deprivation driven by new academic, occupational, and social pressures [10]. The transition to adulthood-including entry into higher education and the labor market and the assumption of family responsibilities-often compromises sleep time, exacerbating previous sleep problems, with distinct patterns between university and non-university students.

Sex differences were also highlighted during this period. Women slept, on average, 42 and 48 minutes longer than men at 18 and 22 years of age, respectively. However, at 22 years of age, they reported poorer sleep quality [10,22]. This disparity may result from the complex interaction of biological, psychological, and sociocultural factors that differentially influence women’s sleep. Biological factors, such as a tendency toward earlier sleep and wake times and hormonal fluctuations across the menstrual cycle and during pregnancy, may both increase and reduce total sleep duration in women [22].

Sociocultural pressures may exacerbate these changes. The transition from adolescence to young adulthood, with changes in marital status, pregnancy, and entry into paid work, among other factors, brings opportunities and demands associated with gender roles [23], such as the double burden. This overload of responsibilities contributes to chronic stress and accumulated mental burden, consequently leading to higher prevalences of insomnia, frequent awakenings, and feelings of fatigue upon waking. Although women sleep longer to compensate for physical and emotional strain, this additional sleep time does not always reverse the effects of these mechanisms, resulting in poorer sleep quality.

These sleep differences, in both women and men, were even more pronounced in racially marginalized groups [23]. Black and Brown women had a higher mean sleep duration than white women, as well as Black and Brown men. These findings reflect how different forms of vulnerability overlap and reinforce one another, disproportionately affecting certain groups. In this sense, it is possible to suggest the simultaneous effects of determinants that amplify racial and gender inequalities [24].

Although several studies, particularly in international contexts, indicate that Black individuals tend to have shorter sleep duration compared to White individuals, the present study found the opposite: the highest mean sleep durations were observed among Black and Brown young individuals [24]. Longer sleep duration may be related to persistent historical inequalities across the life course that limit access to education, employment, health, and other quality resources [25].

In Brazil, unemployment and informality disproportionately affect Black individuals, particularly women [26]. When employment is obtained, insertion conditions are more unfavorable, with greater difficulties in professional advancement and more precarious jobs, and Black men are almost always engaged in manual labor [27]. In education, despite educational expansion since the second half of the twentieth century and advances in affirmative action policies in Brazil, Black individuals present markedly lower educational levels than White individuals [28].

Social determinants such as structural racism, discrimination, economic insecurity, precarious housing, and violence [28,29] directly affect the physical and mental health of these populations, leading to altered sleep patterns. It is important to consider that increased sleep duration may be associated with the exclusion of these groups from social spaces such as higher education or stable employment, and/or with an adaptive response to the chronic stress and adverse conditions they experience. Greater vulnerability to unemployment and engagement in informal occupations may imply greater flexibility or irregularity of schedules, which may also contribute to the longer sleep durations observed in these groups.

Sleep patterns at 18 and 22 years of age reinforce the importance of socioeconomic inequalities. Individuals with lower socioeconomic status reported poorer sleep quality, longer sleep latency, greater fragmentation, and lower efficiency, although findings were less consistent regarding sleep duration [24]. These patterns reflect adverse living conditions (precarious housing, exposure to noise, pollution, and neighborhood insecurity) and demanding work conditions that are often incompatible with adequate sleep (informal work, multiple shifts, or irregular schedules), compounded by chronic psychosocial stress associated with economic precarity.

Although these inequalities frequently overlap with racial and gender issues, socioeconomic factors play a structuring and independent role in sleep patterns. In addition, socioeconomic status may exacerbate gender inequalities. Women in greater economic vulnerability tend to experience even more intense forms of precariousness, in addition to informal and poorly paid work, and lower capacity to control the domestic environment, which amplifies the negative impacts on sleep health [22,24,25].

From a methodological perspective, this study highlights the use of three cohort follow-ups, which allowed the assessment of sleep duration at different ages. The application of robust inequality indicators (SII and MADM) enabled the identification of differences according to sex, race/skin color, and socioeconomic level. Consistent patterns of sleep inequalities were observed, with worse indicators among more vulnerable groups.

Sleep duration was lower at 22 years of age compared to 11 years and higher among females, individuals with lower socioeconomic status, and Black and Brown individuals. At the age of 22, the “very good” sleep quality category was less frequent among females and Brown individuals. These findings reinforce the overlap of social, racial, and gender determinants in shaping sleep patterns and highlight sleep as a marker of health inequities in this population.

## Data Availability

The data used in this study are not available in public repositories due to ethical and confidentiality restrictions, but they may be accessed upon formal request. The database of the 1993 Pelotas Birth Cohort, as well as those of the other cohorts (1982, 2004, and 2015), can be requested by completing a form with an analysis proposal, available at: https://epidemio-ufpel.org.br/coorte-1993/. The request will be submitted for review by a responsible committee. The researchers used ChatGPT (https://chatgpt.com/) to review the grammar in the Discussion section and improve clarity.
