# Supplementary material for: Sociodemographic inequalities in sleep duration and quality at 11, 18, and 22 years of age: a cross-sectional study using data from the 1993 Pelotas Birth Cohort, 2004-2015
Source: Epidemiol Serv Saude. 2026 Aug 5;35:e20260212. doi: 10.1590/S2237-96222026v35e20260212.en (PMC13686403; doi:10.1590/S2237-96222026v35e20260212.en)
Supplement: Translation

# Translation: Desigualdades sociodemográficas na duração e na qualidade do sono aos 11, 18 e 22 anos: estudo transversal com dados da Coorte de Nascimentos de 1993, Pelotas, 2004-2015

**Authors:** Fernando Cesar Wehrmeister, Adriana Kramer Fiala Machado, Angel Caroline Chirivino Antunes da Rocha, Indiara da Silva Viegas, Larissa Adna Neves Silva, Michele Krüger Vaz Moreira, Tainã Dutra Valério, Helen Gonçalves, Ana Maria Baptista Menezes

**Affiliations:** 1 Programa de Pós-graduação em Epidemiologia da Universidade Federal de Pelotas, RS, Brasil Universidade Federal de Pelotas Programa de Pós-graduação em Epidemiologia Universidade Federal de Pelotas RS Brazil; 2 Programa de Pós-graduação em Epidemiologia da Universidade Federal de Pelotas, RS, Brasil Universidade Federal de Pelotas Programa de Pós-graduação em Epidemiologia Universidade Federal de Pelotas RS Brazil; 3 Programa de Pós-graduação em Epidemiologia da Universidade Federal de Pelotas, RS, Brasil Universidade Federal de Pelotas Programa de Pós-graduação em Epidemiologia Universidade Federal de Pelotas RS Brazil; 4 Programa de Pós-graduação em Epidemiologia da Universidade Federal de Pelotas, RS, Brasil Universidade Federal de Pelotas Programa de Pós-graduação em Epidemiologia Universidade Federal de Pelotas RS Brazil; 5 Programa de Pós-graduação em Epidemiologia da Universidade Federal de Pelotas, RS, Brasil Universidade Federal de Pelotas Programa de Pós-graduação em Epidemiologia Universidade Federal de Pelotas RS Brazil; 6 Programa de Pós-graduação em Epidemiologia da Universidade Federal de Pelotas, RS, Brasil Universidade Federal de Pelotas Programa de Pós-graduação em Epidemiologia Universidade Federal de Pelotas RS Brazil; 7 Programa de Pós-graduação em Epidemiologia da Universidade Federal de Pelotas, RS, Brasil Universidade Federal de Pelotas Programa de Pós-graduação em Epidemiologia Universidade Federal de Pelotas RS Brazil; 8 Programa de Pós-graduação em Epidemiologia da Universidade Federal de Pelotas, RS, Brasil Universidade Federal de Pelotas Programa de Pós-graduação em Epidemiologia Universidade Federal de Pelotas RS Brazil; 9 Programa de Pós-graduação em Epidemiologia da Universidade Federal de Pelotas, RS, Brasil Universidade Federal de Pelotas Programa de Pós-graduação em Epidemiologia Universidade Federal de Pelotas RS Brazil

**Keywords:** Desigualdades em Saúde, Sono, Qualidade do Sono, Fatores Socioeconômicos, Estudos Transversais

## Abstract

Objetivo: Analisar a duração e a qualidade do sono em diferentes idades, segundo fatores sociodemográficos. Métodos: Estudo transversal com dados da Coorte de Nascimentos de 1993 de Pelotas, Rio Grande do Sul, com coletas aos 11, 18 e 22 anos. A duração e a qualidade do sono foram autorrelatadas; a duração diária foi estimada a partir dos horários de dormir e acordar em dias úteis, e a qualidade foi avaliada aos 22 anos por meio de uma pergunta sobre a percepção do sono. Foram realizadas análises descritivas e estimadas medidas de desigualdade segundo sexo, raça/cor e índice de bens. Resultados: Foram analisados 4.439, 4.081 e 3.800 indivíduos aos 11, 18 e 22 anos. A média de duração do sono foi 9,7 (intervalo de confiança de 95% - IC95% 9,6; 9,7), 8,4 (IC95% 8,3; 8,5) e 8,0 (IC95% 7,9; 8,0) horas. O índice absoluto de desigualdade aos 18 anos foi -1,3 (IC95% -1,5; -1,0) em mulheres e -0,7 (IC95% -1,0; -0,4) em homens. Aos 22 anos, a proporção de qualidade do sono “muito boa” foi de 19,2% (IC95% 17,4; 21,1) em homens e “muito ruim” foi de 5,2% (IC95% 4,3; 6,2) em mulheres. Conclusão: A duração do sono diminuiu entre os 11 e os 22 anos e foi maior entre mulheres e em grupos mais vulneráveis, nos quais a qualidade do sono muito boa foi menos frequente.

**Table:** Aspectos éticos

Essa pesquisa respeitou os princípios éticos, obtendo os seguintes dados de aprovação:		
Comitê de ética	Número do parecer	Data de aprovação
Universidade Federal de Pelotas	Protocolo nº 29/2003 (11 anos)	1/12/2003
Universidade Federal de Pelotas	Protocolo nº 5/2011 (18 anos)	12/10/2010		
Universidade Federal de Pelotas	1,250,366 (22 anos)	29/9/2015
**Registro de consentimento livre e esclarecido**	Obtido de todos os participantes. O consentimento informado foi obtido de todos os membros ou de seus áis, quando menores de 18 anos	

## Introdução

Alterações no tempo e na qualidade do sono são reconhecidas como problemas relevantes de saúde pública e afetam diretamente o bem-estar físico e mental dos indivíduos [1]. A duração do sono possui um papel importante na manutenção da saúde e influencia mecanismos relacionados ao balanço energético e à produção hormonal, com repercussões tanto na adolescência quanto na vida adulta [2]. Inadequações no sono estão associadas a desfechos negativos em saúde, que incluem obesidade, hipertensão e problemas de saúde mental, como depressão e bipolaridade [3,4].

Ao longo da vida, há tendência à redução do tempo de sono necessário. São recomendadas entre oito e dez horas de sono para adolescentes e entre sete e nove horas para adultos [5]. No entanto, evidências de estudos populacionais entre 2018 e 2023 indicaram que uma parcela considerável não atendia a essas recomendações, variando de 66,0% entre adolescentes a 55,5% entre adultos com 20 anos ou mais, o que caracterizava um cenário de restrição crônica do sono que afeta amplamente diferentes faixas etárias [6,7]. Entre os adultos, observa-se ainda a tendência à redução do tempo de sono ao longo dos anos, principalmente entre indivíduos do sexo masculino [8].

Na adolescência, a duração do sono pode exercer um efeito protetor contra a má qualidade do sono, enquanto o atraso para dormir e um longo período de latência podem estar associados à maior probabilidade de relatos de sono não reparador e insatisfação com o próprio sono [6]. Já na fase adulta, durações de sono mais próximas das recomendações contribuem para melhor função cognitiva e redução do risco de doenças crônicas e neurodegenerativas [2,9].

Além das variações por faixa etária, fatores socio-demográficos também têm sido associados à duração do sono [10,11]. De maneira geral, mulheres dormem mais e relatam pior qualidade do sono do que os homens [10]. Em relação ao nível socioeconômico, adultos brasileiros com maior poder aquisitivo tendem a dormir menos e apresentam melhor qualidade do sono em comparação àqueles com menor nível socioeconômico [12]. Em contraste, em países de alta renda, os mais ricos têm maior duração e qualidade do sono [13]. No contexto brasileiro, são escassos os dados sobre essas associações, com medidas padronizadas, especialmente entre diferentes faixas etárias e ao longo do tempo [14]. Isso torna relevante aprofundar o conhecimento sobre a associação entre aspectos sociodemográficos, a duração e a qualidade do sono em diferentes momentos do ciclo da vida.

Utilizando dados da Coorte de Nascimentos de 1993 de Pelotas, Rio Grande do Sul, o objetivo deste estudo foi analisar a duração e a qualidade do sono em diferentes idades, segundo fatores sociodemográficos.

## Métodos

### Desenho do estudo

Trata-se de estudo transversal com dados da Coorte de Nascimentos de 1993, em Pelotas, Rio Grande do Sul, município de médio porte localizado na região Sul do país. As análises foram realizadas separadamente aos 11, 18 e 22 anos.

### Contexto e participantes

A Coorte de Nascimentos de 1993 incluiu 5.249 indivíduos na etapa perinatal, o que representa 99,7% de todos os nascidos vivos (5.265) de mulheres que residiam na área urbana de Pelotas, no período de 1º de janeiro a 31 de dezembro de 1993. Este estudo utilizou informações coletadas nas etapas dos 11, 18 e 22 anos, realizadas em 2004, 2011 e 2015, com taxas de acompanhamento de 87,5%, 81,4% e 76,3%, respectivamente. Informações mais detalhadas sobre a metodologia de acompanhamento da Coorte podem ser encontradas em outras publicações [15,16].

O tamanho da amostra correspondeu ao número de participantes disponíveis em cada etapa do acompanhamento (11, 18 e 22 anos), considerando apenas indivíduos com informações completas sobre as variáveis de duração e qualidade do sono em cada etapa. A amostra total entrevistada aos 11, 18 e 22 anos foi, respectivamente, de 4.452, 4.106 e 3.810 indivíduos. Após a exclusão de participantes com informações faltantes (13, 25 e 10), as análises incluíram 4.439, 4.081 e 3.800 indivíduos, respectivamente. Aos 18 e aos 22 anos, 31 (0,8%) e 43 (1,1%) participantes estavam grávidas.

### Fontes de dados e mensuração

#### Desfecho

A duração do sono foi definida como a média diária de horas de sono autorreferida nos dias de semana (excluindo os finais de semana), aos 11, 18 e 22 anos. Nos três acompanhamentos, a duração do sono foi avaliada com as seguintes perguntas: “Geralmente, a que horas você dorme em um dia de semana, exceto sábado e domingo?” e “Geralmente, a que horas você acorda em um dia de semana, exceto sábado e domingo?”. A duração do sono foi calculada pela diferença entre o autorrelato de horários de acordar e de dormir durante dias úteis, e avaliada de forma contínua (horas).

A qualidade subjetiva do sono foi avaliada apenas aos 22 anos, com base na percepção geral do sono no último mês, categorizada em muito boa, boa, ruim ou muito ruim.

#### Exposição

As variáveis independentes avaliadas foram: (i) sexo (masculino, feminino), coletado na linha de base;

(ii) raça/cor, autorrelatada aos 15 anos (branca, preta e parda); e (iii) índice de bens (quintis), coletado aos 11, 18 e 22 anos.

O índice de bens baseou-se na análise de componentes principais, considerando alguns bens e as características do domicílio, e foi posteriormente dividido em quintis [17]. O primeiro quintil (Q1) corresponde aos 20% mais pobres da população, enquanto o último quintil (Q5) corresponde aos 20% mais ricos. A variável raça/cor foi categorizada conforme os critérios do Instituto Brasileiro de Geografia e Estatística (brancos, pardos, pretos, amarelos e indígenas). Devido ao tamanho amostral reduzido (3,7%), os indivíduos que se autodeclararam amarelos ou indígenas foram excluídos da análise.

#### Análise estatística

As análises foram conduzidas no software Stata versão 18.0 (Stata Corporation, College Station, Estados Unidos). A descrição da amostra foi realizada por meio da proporção de indivíduos em cada categoria das variáveis sociodemográficas, acompanhada dos respectivos intervalos de confiança de 95% (IC95%). Foi realizada a comparação das proporções entre a amostra analítica e a amostra da linha de base da coorte (perinatal), segundo as variáveis sexo, raça/cor e renda familiar para avaliar possíveis vieses de seleção. A renda familiar foi utilizada nessa comparação devido à falta de informações sobre o índice de bens na linha de base.

A média de duração do sono foi descrita separadamente aos 11, 18 e 22 anos, de acordo com o sexo, o índice de bens e a raça/cor. Foram utilizados os testes t de Student e análise de variância (ANOVA) e, para a variável índice de bens, foi empregado o teste de tendência linear.

Para avaliar possíveis interações na duração do sono, foram utilizados modelos de regressão linear com inclusão de termos de interação entre sexo e raça/cor, e entre sexo e índice de bens, em cada idade. A significância dos termos de interação foi avaliada por meio de testes de Wald (Tabela Suplementar 1).

Diante da evidência de interação, as análises foram conduzidas com dupla estratificação por: (i) sexo e índice de bens; e (ii) sexo e raça/cor. Para avaliar as desigualdades, foi calculado o índice absoluto de

 desigualdades (*slope index inequality* - SII) para a variável de índice de bens [18]. Para a dupla estratificação utilizando a variável de raça/cor, foi calculada a diferença absoluta média em relação à média (*mean absolute difference from the mean* - MADM). Para essa medida de desigualdade, o erro-padrão foi calculado por uma técnica de reamostragem*,* o que permitiu a construção dos intervalos de confiança [19].

### Resultados

A amostra foi composta majoritariamente por indivíduos do sexo feminino nos três acompanhamentos (50,9%, 50,9% e 53,2% aos 11, 18 e 22 anos) e por participantes autodeclarados de raça/cor branca. Aos 11 e aos 22 anos, a distribuição de renda da família diferiu em relação ao período perinatal (p-valor 0,030 e p-valor<0,001). Essa diferença foi principalmente explicada pela menor proporção de indivíduos no segundo quintil (23,3% no perinatal e 20,7% e 20,2% aos 11 e aos 22 anos), enquanto os demais quintis apresentaram valores semelhantes (Tabela Suplementar 2).

A média geral de duração de sono diminuiu com a idade. Aos 11 anos, a média foi 9,7 (IC95% 9,6; 9,7), passando para 8,4 (IC95% 8,3; 8,5) aos 18 anos e para 8,0 horas (IC95% 7,9; 8,0) aos 22 anos.

Mulheres apresentaram maiores médias de duração de sono do que os homens nos dois últimos acompanhamentos (8,8 versus 8,0 aos 18 anos [p-valor<0,001] e 8,3 vs. 7,6 aos 22 anos [p-valor<0,001]). Aos 18 e aos 22 anos, indivíduos autodeclarados brancos apresentaram menor média de duração do sono (18 anos: 8,3; 22 anos: 7,9) em comparação aos pretos (18 anos: 8,7; 22 anos: 8,1) e aos pardos (18 anos: 8,6; 22 anos: 8,2) (Tabela 1). Também foi observado que, quanto maior o nível socioeconômico, menor a duração do sono (p-valor<0,001). No quintil mais pobre, a duração média de horas de sono diminuiu com a idade: 9,8 (IC95% 9,7; 9,9) aos 11 anos, 9,0 (IC95% 8,9; 9,1) aos 18 anos e 8,4 (IC95% 8,2; 8,5) aos 22 anos. No quintil mais rico, os valores da média da duração de sono foram igualmente decaindo: 9,4 (IC95% 9,4; 9,5) horas para os 11 anos, 8,0 (IC95% 7,9; 8,1) horas para os 18 anos e 7,8 (IC95% 7,6; 7,9) horas para os 22 anos (Tabela 1).

Em todos os anos e para ambos os sexos, houve relação inversa entre a duração do sono e os quintis de riqueza, principalmente entre as mulheres a partir dos 18 anos (Figura 1). Nessa mesma idade, o índice absoluto de desigualdades foi de -1,3 hora (IC95% -1,5; -1,0) entre as mulheres e de -0,7 hora (IC95% -1,0; -0,4) entre os homens. As medidas de desigualdade (diferença absoluta média em relação à média) indicaram que as mulheres brancas apresentaram médias de sono menores, com diferenças médias de 0,16 (IC95% 0,08; 0,23) aos 18 anos e de 0,18 (IC95% 0,11; 0,26) aos 22 anos, em relação à média (Figura 2).

Apresentou-se a prevalência das categorias de qualidade do sono aos 22 anos, estratificada por sexo, raça/cor e índice de bens (Tabela 2). A qualidade do sono “muito boa” foi mais frequente entre homens (19,2%; IC95% 17,4; 21,1), enquanto a categoria “muito ruim” foi mais prevalente entre mulheres (5,2%; IC95% 4,3; 6,2). Indivíduos autodeclarados de raça/cor branca apresentaram maior prevalência de qualidade do sono “muito boa” (19,8%; IC95% 18,2; 21,5) em comparação aos autodeclarados pardos (14,0%; IC95% 11,5; 17,0). As prevalências foram semelhantes nas demais categorias de qualidade do sono.

Em relação ao índice de bens, a prevalência de qualidade do sono “muito boa” foi de 14,6% (IC95% 12,3; 17,3) no quintil mais baixo e de 20,3% (IC95% 17,6; 23,3) no mais alto, enquanto a categoria “muito ruim” apresentou prevalências de 4,9% (IC95% 3,6; 6,7) e 3,0% (IC95% 2,0; 4,5). As demais

categorias apresentaram valores semelhantes entre os grupos, com sobreposição dos intervalos de confiança (Tabela 2).

**Tabela 1:** Descrição da amostra, média da duração do sono e intervalo de confiança de 95% (IC95%) segundo sexo, raça/cor e índice de bens aos 11, 18 e 22 anos. Pelotas, 2004 (n=4.439), 2011 (n=4.081) e 2015 (n=3.800)

11 anos (n= 4.439)				18 anos (n=4.081)			22 anos (n=3.800)		
Variáveis	n (%)	Média (IC95%)	p-valor	n (%)	Média (IC95%)	p-valor	n (%)	Média (IC95%)	p-valor
Geral		9,7 (9,6; 9,7)			8,4 (8,3; 8,4)			8,0 (7,9; 8,0)	
Sexo			0,008^b^			<0,001^b^			<0,001^b^
Masculino	2.180 (49,1)	9,6 (9,6; 9,7)		2.004 (49,1)	8,0 (8,0; 8,1)		1.778 (46,8)	7,6 (7,5; 7,7)	
Feminino	2.259 (50,9)	9,7 (9,7; 9,8)		2.077 (50,9)	8,8 (8,7; 8,8)		2022 (53,2)	8,3 (8,2; 8,4)	
Raça/cor^a^			0,944^c^			<0,001^c^			<0,001^b^
Branca	2.704 (63,9)	9,7 (9,6; 9,7)		2.512 (64,2)	8,3 (8,2; 8,4)		2.256 (63,3)	7,9 (7,8; 8,0)	
Preta	601 (14,2)	9,7 (9,6; 9,8)		565 (14,4)	8,7 (8,5; 8,8)		536 (15,0)	8,1 (8,0; 8,3)	
Parda	768 (18,2)	9,7 (9,6; 9,8)		691 (17,7)	8,6 (8,5; 8,7)		635 (17,8)	8,2 (8,1; 8,3)	
Índice de bens (quintis)			<0,001^d^			<0,001^d^			<0,001^d^
Q1 (mais pobre)	859 (20,1)	9,8 (9,7; 9,9)		831 (20,4)	9,0 (8,9; 9,1)		759 (20,0)	8,4 (8,2; 8,5)	
Q2	851 (19,9)	9,8 (9,7; 9,9)		797 (19,5)	8,5 (8,4; 8,6)		759 (20,0)	8,1 (7,9; 8,5)	
Q3	853 (20,0)	9,7 (9,6; 9,8)		816 (20,0)	8,3 (8,2; 8,4)		759 (20,0)	8,0 (7,8; 8,1)	
Q4	854 (20,0)	9,6 (9,5; 9,7)		820 (20,1)	8,2 (8,1; 8,3)		758 (20,0)	7,8 (7,7; 7,9)	
Q5 (mais rico)	856 (20,0)	9,4 (9,4; 9,5)		814 (20,0)	8,0 (7,9; 8,1)		759 (20,0)	7,8 (7,6; 7,9)	

a Variável com maior número de valores faltantes*.* Indivíduos autodeclarados como amarelos e indígenas foram excluídos devido ao tamanho amostral; ^b^Teste t de Student; ^c^Teste de análise de variância (ANOVA); ^d^Teste de tendência linear.

**Figura 1:** Média da duração do sono em horas, índice absoluto de desigualdades (SII) e intervalo de confiança de 95% (IC95%) segundo sexo e índice de bens aos 11, 18 e 22 anos. Pelotas, 2004 (n=4.439), 2011 (n=4.081) e 2015 (n=3.800)

Legenda: Os pontos coloridos representam a média da duração do sono por quintil do índice de bens; as linhas horizontais azuis ilustram a amplitude da desigualdade. O SII expressa a diferença absoluta, em horas, entre os extremos do índice dos bens, considerando toda a distribuição da variável. A duração de sono corresponde à média de horas dormidas durante os dias de semana (segunda-feira a sexta-feira).

**Figura 2:** Média da duração do sono em horas, diferença absoluta média em relação à média (MADM) e intervalo de confiança de 95% (IC95%) segundo o sexo e raça/cor aos 11, 18 e 22 anos. Pelotas, 2004 (n=4.439), 2011 (n=4.081) e 2015 (n=3.800)

Legenda: Os pontos coloridos representam a média da duração do sono de cada grupo de raça/cor; as linhas horizontais azuis ilustram a amplitude da desigualdade. A MADM expressa a dispersão média das categorias em relação à média populacional, em horas. A duração de sono corresponde à média de horas dormidas durante os dias de semana (segunda-feira a sexta-feira).

**Tabela 2:** Prevalência e intervalo de confiança de 95% (IC95%) da qualidade do sono “muito boa”, “boa”, “ruim” e “muito ruim” segundo sexo, raça/cor e índice de bens aos 22 anos. Pelotas, 2015 (n=3.800)

Variáveis	Muito boa		Boa		Ruim		Muito ruim	
	%	IC95%	%	IC95%	%	IC95%	%	IC95%
**Sexo**								
Masculino	19,2	17,4; 21.1	65,2	63,0; 67,4	12,6	11,2; 14,2	3,0	2,3; 3,9
Feminino	17,3	15,7; 19,0	61,7	59,5; 63,8	15,9	14,3; 17,5	5,2	4,3; 6,2
**Raça/cor**								
Branca	19,8	18,2; 21,5	62,0	59,9; 64,0	14,2	12,8; 15,7	4,0	3,3; 4,9
Preta	15,3	12,5; 18,6	65,7	61,5; 69,6	14,2	11,5; 17,4	4,9	3,3; 7,0
Pardo	14,0	11,5; 17,0	66,7	63,0; 70,3	15,5	12,8; 18,5	3,8	2,5; 5,6
**Índice de bens (quintis)**								
Q1 (mais pobre)	14,6	12,3; 17,3	66,5	63,1; 69,8	14,0	11,7; 16,6	4,9	3,6; 6,7
Q2	17,8	15,2; 20,7	62,5	58,9; 65,8	14,6	12,3; 17,3	5,1	3,8; 7,0
Q3	18,6	16,0; 21,5	62,8	59,3; 66,2	14,5	12,2; 17,2	4,1	2,9; 5,7
Q4	19,4	16,8; 22,4	62,4	58,8; 65,7	14,5	12,2; 17,2	3,7	2,6; 5,3
Q5 (mais rico)	20,3	17,6; 23,3	62,5	59,0; 65,9	14,1	11,8; 16,8	3,0	2,0; 4,5

## Discussão

Foram observados padrões consistentes de desigualdades sociodemográficas na duração e na qualidade do sono, com menor duração nas idades mais avançadas e maior duração entre mulheres, entre indivíduos pretos ou pardos e entre aqueles de menor nível socioeconômico. Esses padrões foram mais evidentes aos 18 e aos 22 anos. Em contraste, maiores níveis socioeconômicos estiveram associados à menor duração do sono, particularmente entre mulheres de 18 anos, enquanto as diferenças, segundo a raça/cor, foram mais marcantes entre mulheres adultas.

A interpretação desses achados deve considerar algumas limitações. A estimativa da duração do sono baseada em autorrelato pode ter introduzido viés de informação, o que resultou em possível superestimação da duração do sono, especialmente por não considerar o período de latência e por se basear apenas nos horários de dormir e acordar. Nesse sentido, é possível que os participantes tenham interpretado “dormir” como o momento de se deitar e “acordar” como o de se levantar, o que pode ter inflado o tempo total de sono estimado.

A avaliação restrita aos dias úteis também pode não refletir variações nos padrões de sono nos finais de semana. A ausência de outras dimensões do sono, como latência, variabilidade e sonolência, e de fatores contextuais, como estresse e condições de moradia, responsabilidades familiares e ingresso no ensino superior, limita a compreensão mais abrangente dos mecanismos envolvidos. A indisponibilidade de variáveis, como o nível de atividade física e o excesso de peso, pode restringir a compreensão desses mecanismos, uma vez que podem atuar em etapas intermediárias na relação entre os fatores sociodemográficos e o sono.

A ausência de avaliação consistente da qualidade do sono nos diferentes pontos de seguimento, em contraste com a análise da duração, dificulta a comparação entre as diferentes idades avaliadas e pode obscurecer mudanças relevantes nesse desfecho. Embora a combinação de medidas quantitativas e qualitativas amplie a perspectiva da avaliação, essas limitações devem ser consideradas na interpretação dos resultados, pois podem subestimar a complexidade das desigualdades no sono. Por fim, a exclusão de grupos étnicoraciais minoritários, devido ao pequeno tamanho amostral, pode restringir a análise das desigualdades de forma mais abrangente.

Constitui-se como limitação o uso de referências internacionais baseadas predominantemente em evidências de países de alta renda, que podem não refletir adequadamente as dinâmicas de populações de baixa e média renda [10]. No entanto, a mensuração contínua da duração do sono adotada neste estudo permite uma avaliação mais sensível das diferenças entre grupos sociais.

Os padrões observados podem ser compreendidos à luz da literatura que descreve as mudanças no sono ao longo do desenvolvimento e a influência combinada de fatores biológicos, comportamentais e sociais. A redução na duração do sono entre os 11 e os 18 anos segue uma tendência global bem documentada, influenciada tanto por fatores comportamentais quanto por mudanças biológicas da puberdade [20].

O atraso da fase circadiana, marcado pela secreção tardia de melatonina, desloca a janela ideal de sono para horários mais tardios, o que dificulta que adolescentes adormeçam cedo [2,20]. Nessa faixa etária, esse desalinhamento entre a biologia e as demandas sociais, como os horários escolares, impulsiona despertares e contribui para a privação crônica de sono. O uso noturno de dispositivos eletrônicos intensifica esse quadro, atrasando o ritmo circadiano e agravando a irregularidade do sono [21].

Entre os 18 e os 22 anos, a duração do sono torna-se mais heterogênea e pode apresentar desde a leve recuperação (devido à flexibilidade de horários) até a privação acentuada pelas novas pressões acadêmicas, profissionais e sociais [10]. A transição para a vida adulta - com o ingresso no ensino superior e no mercado de trabalho e a assunção de responsabilidades familiares - frequentemente compromete o tempo de sono, o que agrava problemas anteriores de sono, com padrões distintos entre jovens universitários e não universitários.

Diferenças entre os sexos também se destacaram nesse período. Mulheres dormiram, em média, 42 e 48 minutos a mais do que homens aos 18 e aos 22 anos, respectivamente. No entanto, aos 22 anos, elas relataram pior qualidade de sono [10,22]. Essa disparidade pode decorrer da interação complexa entre fatores biológicos, psicológicos e socioculturais que influenciam, de forma distinta, o sono das mulheres. Fatores biológicos, como a tendência a dormir e acordar mais cedo e as flutuações hormonais ao longo do ciclo menstrual e na gravidez, podem tanto aumentar quanto reduzir o tempo total de sono feminino [22].

Essas alterações podem ser exacerbadas por pressões socioculturais. A transição da adolescência para a juventude, com mudanças no status civil, gravidez e início do trabalho remunerado, entre outros fatores, traz oportunidades e exigências associadas aos papéis de gênero [23], como a dupla jornada de trabalho. Essa sobrecarga de funções contribui para o estresse crônico e a carga mental acumulada, o que, consequentemente, leva a maiores prevalências de insônia, despertares frequentes e sensação de cansaço ao acordar [22]. Embora as mulheres durmam para compensar o desgaste físico e emocional, esse tempo adicional de sono nem sempre reverte os efeitos desses mecanismos, o que resulta em um sono de menor qualidade.

Essas diferenças de sono, tanto em mulheres quanto em homens, foram ainda mais evidentes em grupos racialmente marginalizados [23]. Mulheres pretas e pardas apresentaram maior duração média de sono do que as brancas, e os homens da mesma raça/cor. Tais achados expressaram como diferentes formas de vulnerabilidade se sobrepõem e se potencializam, afetando de forma desproporcional certos grupos. Nesse caso, é possível sugerir que há efeitos simultâneos dos determinantes que potencializam as desigualdades raciais e de gênero [24].

Embora diversos estudos, especialmente em contextos internacionais, apontem que pessoas negras tendem a apresentar menor duração do sono em comparação a pessoas brancas, os resultados deste estudo indicaram o oposto: as maiores médias de duração do sono estavam entre jovens pretos e pardos [24]. A maior duração do sono pode estar relacionada às desigualdades históricas persistentes que permeiam o ciclo de vida dessa população e limitam o acesso a oportunidades de educação, trabalho, saúde e outros recursos qualificados [25].

No Brasil, o desemprego e a informalidade atingem mais pessoas negras, principalmente mulheres [26]. Quando conseguem alguma ocupação, as condições de inserção são mais desfavoráveis, com maiores dificuldades de ascensão profissional e postos de trabalho mais precários, e homens negros quase sempre ocupando trabalhos manuais [27]. Na educação, apesar da expansão educacional desde a segunda metade do século XX e dos avanços na implementação de políticas de ações afirmativas no Brasil, as pessoas negras apresentam níveis de educação marcadamente mais baixos do que as brancas [28].

Determinantes sociais como racismo estrutural, discriminação, insegurança econômica, habitação precária e violência [28,29] afetam diretamente a saúde física e mental dessas populações, o que influencia padrões do sono alterados. É importante considerar que o aumento do tempo de sono pode estar associado à exclusão desses grupos de espaços sociais, como o ensino superior ou o mercado de trabalho estável, e/ou à resposta adaptativa diante do estresse crônico e das condições adversas por eles vivenciadas. A maior vulnerabilidade ao desemprego e à inserção em ocupações informais pode implicar maior flexibilidade ou irregularidade de horários, o que também pode contribuir para maiores durações de sono observadas nesses grupos.

Os padrões de sono aos 18 e aos 22 anos reforçam a importância das desigualdades socioeconômicas. Indivíduos de baixo nível socioeconômico relataram pior qualidade do sono, maior latência para adormecer, maior fragmentação e menor eficiência, embora com resultados menos consistentes quanto à duração do sono [24]. Esses padrões refletem condições de vida adversas (habitações precárias, exposição a ruído, poluição e insegurança nos bairros) e demandas laborais cansativas frequentemente incompatíveis com um sono adequado (trabalhos informais, múltiplos turnos ou horários irregulares), agravadas pelo estresse psicossocial crônico associado à precariedade econômica.

Ainda que essas desigualdades frequentemente se sobreponham às questões raciais e de gênero, as socioeconômicas desempenham papel estruturante e independente sobre os padrões de sono. Além disso, o nível socioeconômico pode agravar as desigualdades de gênero. Mulheres em maior vulnerabilidade econômica tendem a vivenciar formas ainda mais intensas de precariedade, além do trabalho informal e mal remunerado, e menor capacidade de controle sobre o ambiente doméstico, o que potencializa os impactos negativos sobre a saúde do sono [22,24,25].

Do ponto de vista metodológico deste estudo, destacou-se a utilização de três acompanhamentos da Coorte, o que permitiu avaliar a duração do sono em diferentes idades. A aplicação de indicadores robustos de desigualdade (índice absoluto de desigualdades e diferença absoluta média em relação à média) permitiu identificar diferenças por sexo, raça/cor e nível socioeconômico. Observou-se, de forma consistente, a presença de desigualdades nos padrões de sono, com piores indicadores entre os grupos mais vulneráveis.

A duração do sono foi menor aos 22 anos em comparação aos 11 anos e maior entre mulheres, indivíduos de menor nível socioeconômico e pretos e pardos. Aos 22 anos, a pior qualidade do sono “muito boa” foi menos frequente entre mulheres e pardos. Esses achados reforçaram a sobreposição de determinantes sociais, raciais e de gênero na configuração dos padrões de sono e evidenciaram o sono como marcador de iniquidades em saúde nessa população.

## Disponibilidade de dados

Os dados utilizados nesta pesquisa não estão disponíveis em repositórios públicos devido a restrições éticas e de confidencialidade, mas podem ser acessados mediante solicitação formal. O banco de dados da Coorte de Nascimentos de Pelotas de 1993, assim como os das demais coortes (1982, 2004 e 2015), pode ser solicitado mediante o preenchimento de um formulário com proposta de análise, disponível em: https://epidemio-ufpel.org.br/coorte-1993/. A solicitação será submetida à avaliação de uma comissão responsável.

Conflito de interesses

Nenhum declarado.

Uso de inteligência artificial generativa

Os pesquisadores utilizaram o ChatGPT (https://chatgpt.com/) para revisar a gramática da seção *Discussão* e garantir maior clareza.

## References

[1] 1 CHATTU, VK; MANZAR, MD; KUMARY, S; BURMAN, D; SPENCE, DW; PANDI-PERUMAL, SR. The global problem of insufficient sleep and its serious public health implications. Healthcare (Basel). 2018;7(1):E1.10.3390/healthcare7010001PMC647387730577441

[2] 2 KNUTSON, KL; SPIEGEL, K; PENEV, P; VAN CAUTER, E. The metabolic consequences of sleep deprivation. Sleep Med Rev. 2007;11(3):163-178.10.1016/j.smrv.2007.01.002PMC199133717442599

[3] 3 GRIMALDI, M; BACARO, V; NATALE, V; TONETTI, L; CROCETTI, E. The longitudinal interplay between sleep, anthropometric indices, eating behaviors, and nutritional aspects: a systematic review and meta-analysis. Nutrients. 2023;15(14):3179.10.3390/nu15143179PMC1038559637513597

[4] 4 SCOTT, J; KALLESTAD, H; VEDAA, O; SIVERTSEN, B; ETAIN, B. Sleep disturbances and first onset of major mental disorders in adolescence and early adulthood: a systematic review and meta-analysis. Sleep Med Rev. 2021;57(101429):101429.10.1016/j.smrv.2021.10142933549912

[5] 5 HIRSHKOWITZ, M; WHITON, K; ALBERT, SM; ALESSI, C; BRUNI, O; DONCARLOS, L; et al. National Sleep Foundation's updated sleep duration recommendations: final report. Sleep Health. 2015;1(4):233-243.10.1016/j.sleh.2015.10.00429073398

[6] 6 ECHEVARRIA, P; DEL-PONTE, B; TOVO-RODRIGUES, L; MATIJASEVICH, A; HALAL, CS; SANTOS, IS. Screen use and sleep duration and quality at 15 years old: cohort study. Sleep Med X. 2023;5(100073):100073.10.1016/j.sleepx.2023.100073PMC1025106937305851

[7] 7 LIMA, MG; BARROS, MBA; CEOLIM, MF; ZANCANELLA, E; CARDOSO, TAMO. Sleep duration, health status, and subjective well-being: a population-based study. Rev Saude Publica. 2018;52:82.10.11606/S1518-8787.2018052000602PMC612287630183844

[8] 8 ZOMERS, ML; HULSEGGE, G; VAN OOSTROM, SH; PROPER, KI; VERSCHUREN, WMM; PICAVET, HSJ. Characterizing adult sleep behavior over 20 years-the population-based Doetinchem cohort study. Sleep. 2017;40(7).10.1093/sleep/zsx085PMC580524828525637

[9] 9 MEDIC, G; WILLE, M; HEMELS, ME. Short- and long-term health consequences of sleep disruption. Nat Sci Sleep. 2017;9:151-161.10.2147/NSS.S134864PMC544913028579842

[10] 10 MACHADO, AKF; WENDT, A; MENEZES, AMB; GONÇALVES, H; WEHRMEISTER, FC. Sleep duration trajectories from adolescence to emerging adulthood: Findings from a population-based birth cohort. J Sleep Res. 2021;30(3):e13155.10.1111/jsr.1315532808393

[11] 11 WEHRMEISTER, FC; WENDT, A; TAVARES, PS; SANTOS, FSD; DIAS, MS; CARPENA, MX; et al. Short and long sleep duration and associated factors in pre-adolescence and early adulthood: evidence from the 1993 Pelotas birth cohort study. Sleep Med. 2020;75:477-483.10.1016/j.sleep.2020.09.00633007718

[12] 12 BARROS, MBA; LIMA, MG; CEOLIM, MF; ZANCANELLA, E; CARDOSO, TAMO. Quality of sleep, health and well-being in a population-based study. Rev Saude Publica. 2019;53:82.10.11606/s1518-8787.2019053001067PMC676328231576942

[13] 13 SOSSO, FAE; HOLMES, SD; WEINSTEIN, AA. Influence of socioeconomic status on objective sleep measurement: a systematic review and meta-analysis of actigraphy studies. Sleep Health. 2021;7(4):417-428.10.1016/j.sleh.2021.05.00534266774

[14] 14 SIMONELLI, G; MARSHALL, NS; GRILLAKIS, A; MILLER, CB; HOYOS, CM; GLOZIER, N. Sleep health epidemiology in low and middle-income countries: a systematic review and meta-analysis of the prevalence of poor sleep quality and sleep duration. Sleep Health. 2018;4(3):239-250.10.1016/j.sleh.2018.03.00129776618

[15] 15 GONÇALVES, H; ASSUNÇÃO, MC; WEHRMEISTER, FC; OLIVEIRA, IO; BARROS, FC; VICTORA, CG; et al. Cohort profile update: the 1993 Pelotas (Brazil) birth cohort follow-up visits in adolescence. Int J Epidemiol. 2014;43(4):1082-1088.10.1093/ije/dyu077PMC412156024729426

[16] 16 VICTORA, CG; ARAÚJO, CLP; MENEZES, AMB; HALLAL, PC; VIEIRA, MF; NEUTZLING, MB; et al. Methodological aspects of the 1993 Pelotas (Brazil) Birth Cohort Study. Rev Saude Publica. 2006;40(1):39-46.10.1590/s0034-89102006000100008PMC291776716410981

[17] 17 RUSTEIN, SO. The DHS wealth index: approaches for rural and urban areas. Irvine. CA: Macro International Incorporated; 2008 15 maio 2025. https://www.dhsprogram.com/pubs/pdf/WP60/WP60.pdf.

[18] 18 BARROS, AJD; VICTORA, CG. Measuring coverage in MNCH: determining and interpreting inequalities in coverage of maternal, newborn, and child health interventions. PLoS Med. 2013;10(5):e1001390.10.1371/journal.pmed.1001390PMC364621423667332

[19] 19 SCHLOTHEUBER, A; HOSSEINPOOR, AR. Summary measures of health inequality: a review of existing measures and their application. Int J Environ Res Public Health. 2022;19(6):3697.10.3390/ijerph19063697PMC899213835329383

[20] 20 UCCELLA, S; CORDANI, R; SALFI, F; GORGONI, M; SCARPELLI, S; GEMIGNANI, A; et al. Sleep deprivation and insomnia in adolescence: implications for mental health. Brain Sci. 2023;13(4):569.10.3390/brainsci13040569PMC1013668937190534

[21] 21 RICKETTS, EJ; JOYCE, DS; RISSMAN, AJ; BURGESS, HJ; COLWELL, CS; LACK, LC; et al. Electric lighting, adolescent sleep and circadian outcomes, and recommendations for improving light health. Sleep Med Rev. 2022;64(101667):101667.10.1016/j.smrv.2022.101667PMC1069390736064209

[22] 22 CHAO, S-Y; PERELLI-HARRIS, B; BERRINGTON, A; BLOM, N. Sleep hours and quality before and after baby: Inequalities by gender and partnership. Adv Life Course Res. 2023;55(100518):100518.10.1016/j.alcr.2022.10051836942639

[23] 23 PICANÇO, F; ARAÚJO, CMO; SUSSAI, MC. Papéis de gênero e divisão das tarefas domésticas segundo gênero e cor no Brasil: outros olhares sobre as desigualdades. Rev Bras Estud Popul. 2021;38:1-31.

[24] 24 BATOOL-ANWAR, S; QUAN, SF. Sleep health disparity and race/ethnicity, socioeconomic status, and gender: a systematic review. Sleep Med Res. 2024;15(3):139-150.

[25] 25 ECLAC. Social Panorama of Latin America and the Caribbean 2023: labour inclusion as a key axis of inclusive social development. 2023 15 maio 2025. https://repositorio.cepal.org/entities/publication/e71711da-e21c-4070-9836-d3fe5f2c2045.

[26] 26 COBO, B; OLIVEIRA, BMM. Desigualdades no mercado de trabalho brasileiro: uma proposta de conceituação e mensuração do trabalho precário sob a lupa da interseccionalidade. Rev Bras Estud Popul. 2024;41:1-24.

[27] 27 Departamento Intersindical de Estatística e Estudos Socioeconômicos. As dificuldades da população negra no mercado de trabalho. Dieese, 2023 15 maio 2025. https://www.dieese.org.br/boletimespecial/2023/conscienciaNegra2023.html.

[28] 28 MARTELETO, LJ; DONDERO, M. Racial inequality in education in Brazil: a twins fixed-effects approach. Demography. 2016;53(4):1185-1205.10.1007/s13524-016-0484-8PMC502692527443551

[29] 29 MAJENO, A; TSAI, KM; HUYNH, VW; MCCREATH, H; FULIGNI, AJ. Discrimination and sleep difficulties during adolescence: the mediating roles of loneliness and perceived stress. J Youth Adolesc. 2018;47(1):135-147.10.1007/s10964-017-0755-8PMC575008429164378
